# ALZHEIMER’S DISEASE AND DISPARITY: A RURAL UNDERCOUNTING

**DOI:** 10.1093/geroni/igae098.3789

**Published:** 2024-12-31

**Authors:** Noah Brenman, Mengyuan Cheng, Nasim Ferdows

**Affiliations:** Northeastern University, Boston, Massachusetts, United States; Northeastern University, Boston, Massachusetts, United States; Northeastern University, Boston, Massachusetts, United States

## Abstract

Rising life expectancy has led to an increasing prevalence of age-related diseases, particularly Alzheimer’s disease and related dementia. The more rapid increase of the older population in rural areas, combined with a higher prevalence of dementia risk factors, and limited access to healthcare services, may lead to higher rates of dementia in these communities. This project examines the differences in dementia mortality rates between rural and urban areas over a three-year period. We used county-level data on age-adjusted mortality rates from the CDC Wonder Database, merging it with County Health Rankings from 2018-2020. Using Rural-Urban Commuting Area (RUCA) we defined the level of rurality as: Urban, rural-adjacent to an urban area, and rural non-adjacent to an urban area. We found that rural areas had higher dementia age-adjusted death rates compared to urban areas, even after accounting for select county characteristics. The percent difference between rural and urban dementia mortality ranged between 7.1 to 9.8%, showing a persistent widening gap in rural-urban dementia mortality rates.

